# Using Self-Reported Data to Segment Older Adult Populations with Complex Care Needs

**DOI:** 10.5334/egems.275

**Published:** 2019-04-12

**Authors:** Elizabeth A. Bayliss, Jennifer L. Ellis, John David Powers, Wendolyn Gozansky, Chan Zeng

**Affiliations:** 1 Kaiser Permanente Colorado, US; 2 Colorado Permanente Medical Group, US

**Keywords:** cluster analysis, latent class analysis, patient-reported data, multimorbidity

## Abstract

**Background:**

Tailored care management requires effectively segmenting heterogeneous populations into actionable subgroups. Using patient reported data may help identify groups with care needs not revealed in traditional clinical data.

**Methods:**

We conducted retrospective segmentation analyses of 9,617 Kaiser Permanente Colorado members age 65 or older at risk for high utilization due to advanced illness and geriatric issues who had completed a Medicare Health Risk Assessment (HRA) between 2014 and 2017. We separately applied clustering methods and latent class analyses (LCA) to HRA variables to identify groups of individuals with actionable profiles that may inform care management. HRA variables reflected self-reported quality of life, mood, activities of daily living (ADL), urinary incontinence, falls, living situation, isolation, financial constraints, and advance directives. We described groups by demographic, utilization, and clinical characteristics.

**Results:**

Cluster analyses produced a 14-cluster solution and LCA produced an 8-class solution reflecting groups with identifiable care needs. Example groups included: frail individuals with memory impairment less likely to live independently, those with poor physical and mental well-being and ADL limitations, those with ADL limitations but good mental and physical well-being, and those with few health or other limitations differentiated by age, presence or absence of a documented advance directive, and tobacco use.

**Conclusions:**

Segmenting populations with complex care needs into meaningful subgroups can inform tailored care management. We found groups produced through cluster methods to be more intuitive, but both methods produced actionable information. Applying these methods to patient-reported data may make care more efficient and patient-centered.

## Context

Population heterogeneity makes it difficult to design and implement effective care management interventions for individuals with complex care needs [1,2,3]. The most effective programs use multiple modalities to target specific needs or specific subpopulations based on age, diagnoses, or other characteristics [4,5]. However, there is no common or systematic approach to identifying relevant subpopulations.

To address this heterogeneity, the National Academy of Medicine (NAM) has proposed a ‘starter’ typology differentiating 6 groups characterized by age and medical, social, and behavioral needs [6]. Likewise, the American Diabetes Association provides a three-tiered model based on morbidity burden to guide treatment intensity for individuals with diabetes [7]. However, even these typologies are fairly broad. Further, within any delivery system, subgroups of individuals with complex needs may differ. The subpopulations within the Veterans Administration population are likely quite different from those within an urban safety net population, and both of those are likely different from groups within other delivery systems and settings. Delivery systems interested in developing patient-centered care management programs need to understand the characteristics of the subpopulations they serve.

Traditional electronic data such as diagnostic codes and laboratory values may not capture essential information on factors that drive care needs, including function, personal preferences, and social resources, that can only be reported by individuals themselves. Identifying and characterizing complex needs subpopulations requires patient-reported information to help match care delivery to personal needs. Although newer data from electronic health records (EHRs) such as symptom assessments and ICD-10 codes that capture functional status can improve our ability to identify complex needs subpopulations, subjective information can add a level of specificity unlikely to be captured with objective coding.

Using the Medicare Health Risk Assessment, we explored two data-driven methods to segment a heterogeneous population of older adults with potentially complex care needs into clinically meaningful subgroups using self-reported information. The primary purpose of the analysis was to demonstrate how segmentation methods could be applied to patient-reported data, not to generate evidence to inform a taxonomy of subpopulations of older adults. The goals of the segmentation process were 1) to demonstrate the ability to identify groups with unique needs that could inform development of specific care management programs, and 2) to compare the two analytic methods for application to large, diverse populations.

## Case Description

### Population and Setting

The population consisted of Kaiser Permanente Colorado (KPCO) members age 65 and older as of 05/01/2016 who were classified as having advanced illness as of 07/28/2017. Advanced illness was defined as individuals with complex or multiple chronic conditions and geriatric syndromes who are likely to have frequent hospital care needs. Cohort members also must have completed at least one Medicare Health Risk Assessment (HRA) between 05/01/2014 and 04/30/2017. If more than one HRA was administered in this time frame, the latest one was used in analyses. KPCO is a nonprofit integrated delivery system in which most Medicare beneficiaries are enrolled in a Medicare Advantage plan. KPCO’s Institutional Review Board reviewed the project protocol and determined that it did not meet criteria for human subjects’ research and could be reported as operational or quality improvement methods.

### Data Sources and Variables

Input variables for the segmentation analyses were patient-reported variables drawn from the Medicare HRA, a component of the Medicare Annual Wellness Visit designed to identify patient-reported modifiable risk factors and health needs [8]. Required elements include self-assessment of health status, psychosocial risks, depression, behavioral risks, and Activities of Daily Living and Instrumental Activities of Daily Living [9]. Care delivery systems can add additional questions. As illustrated in Table 1, KPCO elected to add questions reflecting other domains. HRAs are completed at or prior to the visit and are addressed at the visit or as part of population care management. HRA responses are stored in extractable fields in the EHR. We dichotomized responses to the HRA questions based on whether the response was likely to prompt a clinical action. For example, if someone reported a fall within the preceding year, this might lead to a referral to physical therapy; or if someone reported a positive response to a depression screening question, that might prompt a referral to mental health services. On the theory that a missing response would not trigger action, we included missing responses as non-trigger responses.

**Table 1:** Potential Cluster/LCA Inputs: HRA Items and Trigger Responses.

Label*	Item	Trigger Response	Non-Trigger Response

GH*	In general, would you say your health is:	Poor, Fair	Good, Very Good, Excellent, Missing.
QoL	In general, would you say your quality of life is:	Poor, Fair	Good, Very Good, Excellent, Missing
PH	In general, how would you rate your physical health?	Poor, Fair	Good, Very Good, Excellent, Missing
MH*	In general, how would you rate your mental health, including your mood and your ability to think?	Poor, Fair	Good, Very Good, Excellent, Missing
Pain*	In the past 7 days, how much did pain interfere with your day to day activities?	Very much, Quite a bit, Somewhat	A little bit, Not at all, Missing
Sleep*	During the past month, how would you rate your sleep quality overall?	Very bad, Fairly bad	Fairly good, Very good. Missing
PHQ-2*	Little interest or pleasure in doing things Feeling down, depressed, or hopeless	Sum score of 3 or higher: Not at all (0), Several days (1), More than half the days (2), Nearly every day	Sum score of <3: Not at all (0), Several days (1), More than half the days (2), Nearly every day
GAD-2*	Feeling anxious, nervous or on edge Not being able to stop or control worrying	Sum score of 3 or higher: Not at all (0), Several days (1), More than half the days (2), Nearly every day	Sum score of <3: Not at all (0), Several days (1), More than half the days (2), Nearly every day
Angry	In the past 7 days, how often did you feel angry?	Always, Often	Sometimes, Rarely, Never, Missing
Lonely*	How often do you feel lonely or isolated from those around you?	Always, Often	Sometimes, Rarely, Never, Missing
Fall*	Did you fall within the past 12 months?	Yes	No, Missing
Balance*	In the past 12 months, have you had a problem with balance or walking?	Yes	No, Missing
Hearing*	Do you think you have a hearing problem or do others think you have a hearing problem?	Yes	No, Missing
Tooth/Mouth*	Do you have tooth or mouth problems that make it hard for you to eat?	Yes	No, Missing
UI*	In the past 6 months, have you accidentally leaked urine?	Yes	No, Missing
Memory*	In the last year, have you or any of your friends or family felt concerned about any changes in your memory, attention, language skills, or thinking?	Yes	No, Missing
Bathing	Because of a health or physical problem, do you have any difficulty with bathing without help or special equipment?	Need help or special equipment, Do myself with some difficulty	Do myself with no difficulty
Dressing*	Because of a health or physical problem, do you have any difficulty with dressing without help or special equipment?	Need help or special equipment, Do myself with some difficulty	Do myself with no difficulty
Toilet	Because of a health or physical problem, do you have any difficulty with using the toilet without help or special equipment?	Need help or special equipment, Do myself with some difficulty	Do myself with no difficulty
Bed/Chairs	Because of a health or physical problem, do you have any difficulty with getting in and out of bed/chairs without help or special equipment?	Need help or special equipment, Do myself with some difficulty	Do myself with no difficulty
Eating*	Because of a health or physical problem, do you have any difficulty with eating without help or special equipment?	Need help or special equipment, Do myself with some difficulty	Do myself with no difficulty
Taking Meds*	Because of a health or physical problem, do you have any difficulty with taking your medicines without help or special equipment?	Need help or special equipment, Do myself with some difficulty	Do myself with no difficulty
Money	Because of a health or physical problem, do you have any difficulty with managing your money without help or special equipment?	Need help or special equipment, Do myself with some difficulty	Do myself with no difficulty
Household	Because of a health or physical problem, do you have any difficulty with household activities without help or special equipment?	Need help or special equipment, Do myself with some difficulty	Do myself with no difficulty
Shopping*	Because of a health or physical problem, do you have any difficulty with shopping for groceries, etc. without help or special equipment?	Need help or special equipment, Do myself with some difficulty	Do myself with no difficulty
Tobacco*	Do you use any kind of tobacco?	Yes	No, Missing
Physically Inactive*	Combination of days per week and minutes per day of moderate exercise.	0–38 minutes per week	Missing, >38 minutes per week
Fewer than 2 Meals	Do you eat fewer than 2 meals a day?	Yes	No, Missing
Money for Food	Do you always have enough money to buy the food you need?	No	Yes, Missing
Alcohol	Combination of gender, days per week, and drinks per day	Males: ≥15 drinks per week Females: ≥8 drinks per week	Males: <15 drinks per week Females <8 drinks per week Missing
Advance Directive*	Do you have any advance directives for your health care?	No	Yes, Missing
Proxy	Who provided the answers to these questions?	Person to whom the questionnaire was addressed with help from another person; Family member, friend, caregiver	Person to whom the questionnaire was addressed without help from another person

* Indicates variables used as final cluster inputs.

Iterative variable simplification is a key element of exploratory cluster analyses. Prior to running segmentation analyses, we examined all HRA items and removed those less likely to define actionable subpopulations based on clinical judgment (e.g., daily servings of fruits and vegetables), and those that were strongly associated with others (e.g., general health and physical health or difficulty dressing, toileting, bathing, and getting in and out of bed/chairs). After examining initial clusters, we removed additional inputs that did not contribute to defining clusters, such as those with low prevalence (e.g., poor quality of life or anger). Variables and domains that are included in the KPCO HRA but were eliminated during the variable reduction process include quality of life, physical health, anger, difficulty bathing, difficulty toileting, difficulty getting in and out of bed/chairs, difficulty managing money, difficulty with household activities, eating fewer than 2 meals/day, having enough money for food, and alcohol use. Table 1 lists all initially considered variables, noting which were included in the final segmentation analyses along with dichotomized ‘trigger’ responses.

After segmentation based on patient-reported HRA variables, additional clinical, care delivery, and demographic variables were used to describe population segments. These variables were drawn from the HRA and from KPCO’s Virtual Data Warehouse (VDW), a quality-controlled data repository including health care utilization, diagnoses, demographics, and enrollment. Utilization variables included emergency department (ED) visits, inpatient admissions, and observation admissions. We also described population segments by demographic (age, gender, education, marital status, independent living) and clinical (Quan Elixhauser score, [10] cancer history) characteristics. These variables were selected based on their potential to explain the clusters that had been derived from the patient-reported data, but that could have been less useful in segmenting this particular population if included as input variables. For example, adding ED utilization as an input variable could result in clusters of individuals with higher and lower ED use, but might not capture the difference between ED use for pain vs. ED use for falls.

Variable selection (both input variables and descriptive variables) for clustering methods is highly dependent on the population, available data, and planned application of the results. Because clustering is a method for exploring data and populations rather than generating evidence, variable selection is highly iterative and can be revised as more or less actionable clusters are identified.

### Analytic Approach

We used both cluster and latent class analyses to identify relatively homogeneous subgroups within the heterogeneous cohort of older adults. Input variables listed in Table 1 were used in these analyses.

#### Cluster analysis

Cluster analysis refers to classification methods used for discovering groups or “clusters” of highly similar entities within data sets so that observations within one group are as like each other as possible and as dissimilar as possible to observations in all other groups.

Due to the large size of our data set, we used a combined hierarchical and partitive method of generating clusters. We first used k-means clustering (PROC FASTCLUS) to generate a large number of primary clusters and saved the centroids; then we used hierarchical clustering (Ward’s method) on these centroids to determine the recommended number of clusters based on the Cubic Clustering Criterion (CCC) and the pseudo-F statistic (PSF). The recommended number of clusters was then specified as seeds in the k-means clustering to group all the observations. The cluster analysis was implemented using SAS™ software version 9.4, SAS Institute, Cary, NC, USA.

#### Latent class analysis (LCA)

Latent class analysis is a method to identify underlying latent (unobserved) classes (LC) of people using individual level observed variables. Each LC represents a subgroup of individuals characterized by a pattern of responses on a set of categorical input variables. LCA was implemented using poLCA package in R with the same dichotomous variables as in Cluster analysis [11]. We examined multiple LCA models with 1–10 class solutions, and the best fitting model was determined with the smallest Bayesian information criterion (BIC) and clinical interpretability. The chosen algorithm uses a finite mixture model and finds maximum likelihood estimates of model parameters with expectation-maximization and Newton-Raphson methods [11].

Finally, we evaluated the results of the cluster and LCA analyses. We expected that different segmentation methods would yield different groups and different numbers of groups and could potentially lead to different interpretations [12]. The goal of both methods in this context was to identify clinically or operationally meaningful population segments, and to see whether the two methods identified common subgroups.

## Findings

Of 20,316 older adults classified as having advanced illness and potential complex care needs, 9,617 completed at least one HRA during the project period and comprised the analytic cohort for the segmentation analyses. HRA completers were marginally older and healthier than non-completers and had a longer enrollment duration (data not shown). Characteristics of the analytic cohort are provided in the first column of Table 2.

**Table 2:** Cohort description and example clusters.^1^

	Total Sample		Example Clusters				

	N = 9617	%	A	B	C	D	E

Input Variable			N = 278 (2.9%)^2^	N = 2107 (21.9%)^2^	N = 515 (5.3%)^2^	N = 345 (3.6%)^2^	N = 408 (4.2%)^2^

Fair or Poor General/Physical Health	1,599	16.6%	**230(82.7)**	*74(3.5)*	162(31.5)	**242(70.1)**	106(26.0)
Fair or Poor Mental Health	937	9.7%	**149(53.6)**	*44(2.1)*	*9(1.7)*	**329(95.4)**	32(7.8)
Positive on PHQ-2	887	9.2%	**215(77.3)**	*45(2.1)*	41(8.0)	**202(58.6)**	43(10.5)
Positive on GAD-2	240	2.5%	**59(21.2)**	*12(0.6)*	7(1.4)	**202(58.6)**	6(1.5)
Pain Interferes with Activities	3,567	37.1%	**259(93.2)**	*274(13.0)*	**504(97.9)**	241(69.9)	**400(98.0)**
Fairly or Very Bad Sleep Quality	1,617	16.8%	**194(69.8)**	*126(6.0)*	120(23.3)	166(48.1)	**408(100.0)**
Often or Always Lonely/Isolated	276	2.9%	**60(21.6)**	*14(0.7)*	8(1.6)	**75(21.7)**	14(3.4)
Fall in Past 12 Months	2,407	25.0%	**158(56.8)**	*217(10.3)*	95(18.4)	112(32.5)	67(16.4)
Problem with Balance or Walking	3,984	41.4%	**258(92.8)**	*63(3.0)*	425(82.5)	226(65.5)	128(31.4)
Problems with Hearing	4,468	46.5%	**181(65.1)**	*683(32.4)*	237(46.0)	220(63.8)	190(46.6)
Tooth/Mouth Problems	1,002	10.4%	**102(36.7)**	*64(3.0)*	*0(0.0)*	45(13.0)	38(9.3)
Accidentally Leaked Urine	4,244	44.1%	**209(75.2)**	*544(25.8)*	342(66.4)	170(49.3)	194(47.5)
Problems with Memory	1,670	17.4%	145(52.2)	*88(4.2)*	38(7.4)	190(55.1)	40(9.8)
Difficulty Dressing/Using Toilet/Bathing/Getting In & Out of Bed	1,008	10.5%	**197(70.9)**	*28(1.3)*	24(4.7)	25(7.2)	23(5.6)
Difficulty Eating	371	3.9%	**89(32.0)**	*12(0.6)*	9(1.7)	8(2.3)	9(2.2)
Difficulty Taking Medicines/Managing $	496	5.2%	**74(26.6)**	*8(0.4)*	8(1.6)	18(5.2)	12(2.9)
Difficulty Shopping/Household Activities	1,903	19.8%	**260(93.5)**	*0(0.0)*	131(25.4)	50(14.5)	55(13.5)
Tobacco Use	628	6.5%	27(9.7)	*80(3.8)*	36(7.0)	28(8.1)	25(6.1)
Physically Inactive	3,139	32.6%	**225(80.9)**	*0(0.0)*	**512(99.4)**	151(43.8)	70(17.2)
Not Always Enough Money for Food	368	3.8%	**44(15.8)**	40(1.9)	18(3.5)	32(9.3)	13(3.2)
Do Not Live Independently	2,222	23.1%	62(22.3)	*0(0.0)*	130(25.2)	81(23.5)	98(24.0)
No Advance Directive	2,916	30.3%	104(37.4)	*0(0.0)*	163(31.7)	137(39.7)	147(36.0)
**Descriptive variable**							

Education							
<HS Graduate	703	7.3%	40(14.4)	81(3.8)	46(8.9)	45(13.0)	36(8.8)
HS/Some College	4,868	50.6%	166(59.7)	918(43.6)	314(61.0)	188(54.5)	217(53.2)
College Graduate or More	3,349	34.8%	47(16.9)	898(42.6%)	133(25.8%)	91(26.4%)	133(32.6%)
Missing	697	7.2%	25(9.0)	210(10.0)	22(4.3)	21(6.1)	22(5.4)
Marital Status							
Married/Committed Relationship	5,537	57.6%	138(49.6)	1224(58.1)	267(51.8)	186(53.9)	247(60.5)
Single/Divorced/Separated/Widowed	3,478	36.2%	121(43.5)	692(32.8)	230(44.7)	141(40.9)	144(35.3)
Missing	602	6.3%	19(6.8)	191(9.1)	18(3.5)	18(5.2)	17(4.2)
History of cancer	2,836	29.5%	63(22.7)	706(33.5)	146(28.3)	85(24.6)	116(28.4)
Age at MTHA							
63–69	2,273	23.6%	59(21.2)	520(24.7)	95(18.4)	102(29.6)	131(32.1)
70–79	4,478	46.6%	128(46.0)	1072(50.9)	250(48.5)	153(44.3)	196(48.0)
80+	2,866	29.8%	91(32.7)	515(24.4)	170(33.0)	90(26.1)	81(19.9)
Female gender	5,043	52.4%	171(61.5)	1031(48.9)	347(67.4)	168(48.7)	210(51.5)
Quan Elixhauser score							
Mean(SD)	4.3 ± 2.7		5.8 ± 2.6	3.6 ± 2.5	5.1 ± 2.7	5.1 ± 2.6	4.6 ± 2.8
Median(25%, 75%)	4(0–17)		6.0(0–13)	3.0(0–14)	5.0(0–16)	5.0(0–13)	4.0(0–15)
Hospital utilization							
ED Visit(s) 0/1	2,029	21.1%	88(31.7)	373(17.7)	119(23.1)	98(28.4)	91(22.3)
Inpatient Admission(s) 0/1	1,933	20.1%	60(21.6)	351(16.7)	131(25.4)	67(19.4)	100(24.5)
Observation Admission(s) 0/1	833	8.7%	35(12.6)	150(7.1)	42(8.2)	41(11.9)	38(9.3)

^1^ **Bold** shading indicates cluster has a proportion of the input variable that is greater than the 95% CI for the population average and the 1^st^ or 2^nd^ highest proportion of all clusters. *Italics* shading indicates the cluster has a proportion of the input variable that is less than the 95% CI for the population average and the 1^st^ or 2^nd^ lowest proportion of all clusters. ^2^ Row percentages this row only. Row percentages to do not add to 100% as these are selected example clusters. All other percentages in the table reflect proportions of columns.

In the cluster analysis, we selected a 14-cluster solution as one that was manageable, corresponded to peaks in the CCC and PSF values, and seemed likely to segment the cohort into clinically actionable subgroups. Table 2 provides characteristics of the overall analytic cohort and selected illustrative clusters and the full 14-cluster solution is presented in Appendix A. In this application, the analysis identified smaller clusters of individuals who reported poor physical and/or emotional health with or without functional limitations (Clusters A and D), as well as a larger cluster of individuals reporting better health status (Cluster B). The analysis also identified clusters characterized by discrete health needs such as reported problems with pain, balance and walking, and inactivity (Cluster C), and by pain and poor sleep quality without inactivity (Cluster E).

Descriptions of clusters by morbidity and utilization during the project period generally reflected the self-reported data, with Cluster A characterized by high morbidity burden and hospital utilization, Cluster D by higher morbidity burden and emergency service use, and Cluster B by lower morbidity and utilization. Cluster C, in which HRA responses highlighted pain and inactivity, was also characterized by higher hospital utilization.

**Figure 1:**
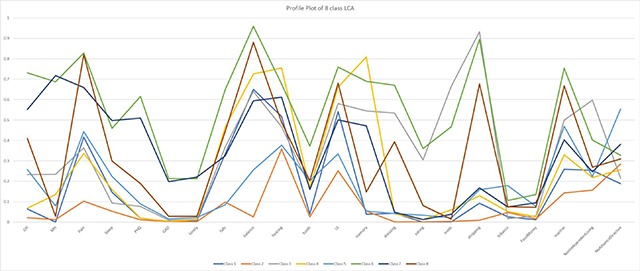
Schematic of latent class analysis results: 8 classes.

The LCA analysis produced an 8-class solution reflecting subgroups with different patterns of variable combinations (Figure 1, Table 3). In these illustrative subgroups, class 2 demonstrates consistently low probabilities of trigger responses to the patient-reported variables—indicating a class with lower morbidity and higher function, while class 6 demonstrates higher probabilities of trigger responses indicating a subgroup of higher morbidity and lower functioning. As with the cluster analyses, the LCA analysis revealed a large subgroup of relatively lower morbidity and several smaller subgroups of individuals reporting either global or specific health concerns.

**Table 3:** Latent class analysis results: 8 Classes.

	Total	1	2	3	4	5	6	7	8

	N = 9617 (%)	N = 2025 (%)	N = 3848 (%)	N = 315 (%)	N = 610 (%)	N = 1007 (%)	N = 318 (%)	N = 475 (%)	N = 1019 (%)

Fair or Poor General Health	1599(16.6)	*104(5.1)*	*59(1.5)*	72(22.9)	34(5.6)	356(35.4)	**239(75.2)**	**278(58.5)**	457(44.8)
Fair or Poor Physical Health	2098(21.8)	*286(14.1)*	*167(4.3)*	96(30.5)	*86(14.1)*	370(36.7)	**246(77.4)**	**300(63.2)**	547(53.7)
Fair or Poor Mental Health	937(9.7)	*0(0.0)*	*45(1.2)*	80(25.4)	84(13.8)	91(9.0)	**237(74.5)**	**382(80.4)**	18(1.8)
Positive on PHQ-2	887(9.2)	37(1.8)	*42(1.1)*	22(7.0)	*6(1.0)*	101(10.0)	**200(62.9)**	**269(56.6)**	210(20.6)
Positive on GAD-2	240(2.5)	*6(0.3)*	*8(0.2)*	2(0.6)	*1(0.2)*	17(1.7)	**70(22.0)**	**108(22.7)**	28(2.7)
Pain Interferes with Activities	3567(37.1)	914(45.1)	*405(10.5)*	*106(33.7)*	208(34.1)	492(48.9)	**266(83.6)**	319(67.2)	**857(84.1)**
Fairly or Very Bad Sleep Quality	1617(16.8)	322(15.9)	*201(5.2)*	*26(8.3)*	100(16.4)	264(26.2)	**149(46.9)**	**243(51.2)**	312(30.6)
Often or Always Lonely/Isolated	276(2.9)	*12(0.6)*	*11(0.3)*	4(1.3)	6(1.0)	26(2.6)	**72(22.6)**	**117(24.6)**	28(2.7)
Fall in Past 12 Months	2407(25.0)	747(36.9)	*377(9.8)*	113(35.9)	**286(46.9)**	*56(5.6)*	**214(67.3)**	151(31.8)	463(45.4)
Problem with Balance or Walking	3984(41.4)	1605(79.3)	*0(0.0)*	198(62.9)	466(76.4)	*200(19.9)*	**309(97.2)**	283(59.6)	**923(90.6)**
Problems with Hearing	4468(46.5)	**1099(54.3)**	*1399(36.4)*	147(46.7)	**454(74.4)**	*360(35.7)*	**218(68.6)**	295(62.1)	496(48.7)
Tooth/Mouth Problems	1002(10.4)	*79(3.9)*	*94(2.4)*	56(17.8)	97(15.9)	**264(26.2)**	**122(38.4)**	70(14.7)	220(21.6)
Accidentally Leaked Urine	4244(44.1)	1138(56.2)	*993(25.8)*	180(57.1)	416(68.2)	*326(32.4)*	**243(76.4)**	239(50.3)	**709(69.6)**
Problems with Memory	1670(17.4)	*3(0.1)*	231(6.0)	179(56.8)	**596(97.7)**	*45(4.5)*	**229(72.0)**	240(50.5)	147(14.4)
Difficulty Dressing	1008(10.5)	88(4.3)	*3(0.1)*	**169(53.7)**	*23(3.8)*	45(4.5)	**219(68.9)**	19(4.0)	442(43.4)
Difficulty Using the Toilet	618(6.4)	*49(2.4)*	*36(0.9)*	**115(36.5)**	21(3.4)	37(3.7)	**115(36.5)**	23(4.8)	190(18.6)
Difficulty Bathing	1311(13.6)	*139(6.9)*	*78(2.0)*	**187(59.4)**	64(10.5)	107(10.6)	**233(73.3)**	68(14.3)	435(42.7)
Difficulty Getting In and Out of Bed/Chairs	1484(15.4)	267(13.2)	*73(1.9)*	**154(48.9)**	103(16.9)	*83(8.2)*	**233(73.3)**	84(17.7)	487(47.8)
Difficulty Eating	371(3.9)	*0(0.0)*	*4(0.1)*	**106(33.7)**	8(1.3)	42(4.2)	**118(37.1)**	4(0.8)	89(8.7)
Difficulty Taking Medicines	496(5.2)	*0(0.0)*	*11(0.3)*	**245(77.8)**	36(5.9)	24(2.4)	**155(48.7)**	17(3.6)	8(0.8)
Difficulty Managing Money	805(8.4)	*64(3.2)*	*58(1.5)*	**83(58.1)**	82(13.4)	64(6.4)	**172(54.1)**	69(14.5)	113(11.1)
Difficulty with Household Activities	2177(22.6)	*320(15.8)*	*119(3.1)*	**274(87.0)**	123(20.2)	222(22.0)	**285(89.6)**	134(28.2)	700(68.7)
Difficulty Shopping	1903(19.8)	*204(10.1)*	*22(0.6)*	**301(95.6)**	74(12.1)	177(17.6)	**291(91.5)**	73(15.4)	761(74.7)
Tobacco Use	628(6.5)	*48(2.4)*	174(4.5)	*4(1.3)*	*30(4.9)*	**231(22.9)**	**34(10.7)**	34(7.2)	73(7.2)
Physically Inactive	3139(32.6)	*534(26.4)*	*549(14.3)*	152(48.3)	197(32.3)	556(55.2)	**247(77.7)**	184(38.7)	**720(70.7)**
Not Always Enough Money for Food	368(3.8)	*18(0.9)*	*56(1.5)*	10(3.2)	18(3.0)	**99(9.8)**	**45(14.2)**	**47(9.9)**	75(7.4)
Do Not Live Independently	2222(23.1)	532(26.3)	*619(16.1)*	**197(62.5)**	139(22.8)	218(21.6)	**128(40.3)**	118(24.8)	271(26.6)
No Advance Directive	2916(30.3)	*367(18.1)*	1082(28.1)	*68(21.6)*	146(23.9)	**659(65.4)**	108(34.0)	**173(36.4)**	313(30.7)
**Descriptive variables**									

Education									
<HS Graduate	703(7.3)	100(4.9)	207(5.4)	24(7.6)	44(7.2)	127(12.6)	47(14.8)	67(14.1)	87(8.5)
HS/Some College	4868(50.6)	990(48.9)	1845(47.9)	124(39.4)	309(50.7)	584(58.0)	174(54.7)	256(53.9)	586(57.5)
College Graduate or More	3349(34.8)	831(41.0)	1542(40.1)	82(26.0)	226(37.0)	213(21.2)	62(19.5)	124(26.1)	269(26.4)
Missing	697(7.2)	104(5.1)	254(6.6)	85(27.0)	31(5.1)	83(8.2)	35(11.0)	28(5.9)	77(7.6)
Marital Status									
Married/Committed Relationship	5537(57.6)	1181(58.3)	2426(63.0)	115(36.5)	360(59.0)	538(53.4)	140(44.0)	256(53.9)	521(51.1)
Single/Divorced/Separated/Widowed	3478(36.2)	751(37.1)	1201(31.2)	124(39.4)	223(36.6)	398(39.5)	150(47.2)	194(40.8)	437(42.9)
Missing	602(6.3)	93(4.6)	221(5.7)	76(24.1)	27(4.4)	71(7.1)	28(8.8)	25(5.3)	61(6.0)
History of cancer	2836(29.5)	601(29.7)	1206(31.3)	73(23.2)	184(30.2)	267(26.5)	69(21.7)	130(27.4)	306(30.0)
Age at MTHA									
63–69	2273(23.6)	377(18.6)	1053(27.4)	44(14.0)	99(16.2)	324(32.2)	61(19.2)	137(28.8)	178(17.5)
70–79	4478(46.6)	920(45.4)	1936(50.3)	99(31.4)	280(45.9)	465(46.2)	136(42.8)	212(44.6)	430(42.2)
80+	2866(29.8)	728(36.0)	859(22.3)	172(54.6)	231(37.9)	218(21.6)	121(38.1)	126(26.5)	411(40.3)
Female gender	5043(52.4)	1098(54.2)	1822(47.3)	199(63.2)	307(50.3)	520(51.6)	194(61.0)	234(49.3)	669(65.7)
Quan Elixhauser score									
Mean(SD)	4.3 ± 2.7	4.4 ± 2.7	3.7 ± 2.5	5.2 ± 2.9	4.5 ± 2.6	4.5 ± 2.8	5.7 ± 2.7	4.9 ± 2.6	5.4 ± 2.9
Median(25%, 75%)	4(2–6)	4(2–6)	3(2–5)	5(3–7)	4(2–6)	4(2–6)	6(4–7)	5(3–7)	5(3–7)
Hospital utilization									
ED Visit(s) 0/1	2029(21.1)	417(20.6)	659(17.1)	91(28.9)	166(27.2)	198(19.7)	103(32.4)	123(25.9)	272(26.7)
Inpatient Admission(s) 0/1	1933(20.1)	455(22.5)	646(16.8)	89(28.3)	126(20.7)	192(19.1)	63(19.8)	91(19.2)	271(26.6)
Observation Admission(s) 0/1	833(8.7)	205(10.1)	248(6.4)	36(11.4)	44(7.2)	89(8.8)	48(15.1)	50(10.5)	113(11.1)

**Bold** shading indicates cluster has a proportion of the input variable that is greater than the 95% CI for the population average and the 1^st^ or 2^nd^ highest proportion of all clusters. *Italics* shading indicates the cluster has a proportion of the input variable that is less than the 95% CI for the population average and the 1^st^ or 2^nd^ lowest proportion of all clusters.

In some cases, clustering analysis and LCA seemed to identify common subgroups. For instance, 91 percent of cluster D was also in class 7; both the cluster and latent class were characterized by poor physical and mental health. In other cases, individuals in a given cluster had approximately equal representation in two or more latent classes; cluster E (pain and poor sleep quality) was associated with both latent class 1 and latent class 5, neither of which had high probabilities of pain or poor sleep quality.

## Major Themes

The NAM describes the first key requirement in caring for high needs patients as segmenting patients based on factors that drive health care [6]. This application of cluster and latent class analyses illustrates that both methods can be used to segment heterogeneous populations into clinically meaningful subgroups. Further, when these methods are applied to systematically collected patient-reported data, they may produce subgroups that better capture subjective care needs. Subjective information can supplement traditional administrative data on utilization and diagnoses to identify subgroups that are clinically actionable and can better inform clinical care management than traditional administrative data alone.

Some segments elicited through LCA analyses appeared to have similar characteristics to those created through clusters. Although both methods can be used to segment heterogeneous clinical populations, cluster analyses create subgroups characterized by more ‘all or nothing’ categories than LCA analyses and may be more clinically interpretable and exhaustive in finding groups. Alternatively, LCA identified primary subpopulations using fewer groups, and groups could be easily represented graphically (Figure 3). Both methods require iterative interpretation to develop a meaningful set of subgroups, and neither method will produce subgroups that are all actionable. It is possible that both methods may perform differently in different data sets. Other approaches such as predictive modeling may also be useful for segmenting complex populations using large clinical data sets [13]. Additional comparisons between these and other methods may help delineate which perform best in specific settings [12].

The Medicare HRA is designed to help clinicians address patient-reported risks for preventable adverse outcomes. Although the HRA is most commonly applied at the point of care, if data are systematically collected, representative, and stored in extractable formats, they can be used to inform program development, population health, and outcomes research [14]. Although content collected through patient-reported outcomes may duplicate content obtainable through more traditional clinical data such as ICD codes, ICD codes alone are unlikely to capture subjective responses to questions about pain, loneliness, and independent activities of daily living (for example). In this project, HRA data revealed meaningful subgroups that might not have been obvious from other electronic clinical data and could inform specific clinical interventions. Important differentiators included function, falls, perceived health status, emotional well-being, pain, and presence or absence of an advance directive. Two large subgroups comprised relatively healthy individuals who could benefit from watchful waiting and routine preventive care plus (for one group) life care planning. Much smaller subgroups could be targeted for more intensive and tailored care management. The size of these subgroups can inform resource allocation within delivery systems.

Utilization and cost of care are often primary concerns for patients with complex care needs, their clinicians, and delivery systems. However, using utilization as a target criterion for care management can miss patients who report high needs but may not (yet) be using significant resources. For example, all our clusters had relatively equal proportions of patients enrolled in an internal utilization-based care management program. This suggests that utilization does not identify all individuals with care needs and that using patient-reported data may be able to identify individuals at risk prior to incurring higher costs of care.

## Limitations

This project was designed to apply exploratory segmentation methods to systematically collected, patient-reported data. It was not designed to generate evidence on caring for specific subgroups. The analytic cohort was neither representative of the KPCO Medicare population nor of Medicare beneficiaries elsewhere, but rather reflected a convenience sample for whom HRA data were available. Therefore, the specific subgroups illustrate differences within the analytic cohort, but are not themselves generalizable. The characteristics of subgroups may not apply to other populations. In addition, the KPCO HRA is not a comprehensive assessment of all complex needs, although it addresses essential domains that predict care needs and quality of life.

## Conclusions

The value of segmentation methods depends on the quality and representativeness of the input data. Using patient-reported data to inform population-level care design and delivery will require a cultural and resource shift towards prioritizing patient-reported data collection and use. Segmentation methods can be used alone or combined with predictive models to identify clinically actionable subgroups and inform care for heterogeneous populations with substantial and varied care needs [15].

## Additional File

The additional file for this article can be found as follows:

10.5334/egems.275.s1 Appendix A. Full 14 cluster solution.

## References

[1] Peikes, D, Chen, A, Schore, J and Brown, R. Effects of care coordination on hospitalization, quality of care, and health care expenditures among Medicare beneficiaries: 15 randomized trials. Jama. 2009; 301(6): 603–618. DOI: 10.1001/jama.2009.126 19211468

[2] Anderson, GF, Ballreich, J, Bleich, S, et al. Attributes common to programs that successfully treat high-need, high-cost individuals. Am J Manag Care. 2015; 21(11): e597–e600. 26735292

[3] Boult, C, Reider, L, Leff, B, et al. The effect of guided care teams on the use of health services: Results from a cluster-randomized controlled trial. Archives of internal medicine. 2011; 171(5): 460–466. DOI: 10.1001/archinternmed.2010.540 21403043 PMC4450357

[4] Brown, RS, Peikes, D, Peterson, G, Schore, J and Razafindrakoto, CM. Six features of Medicare coordinated care demonstration programs that cut hospital admissions of high-risk patients. Health Affairs. 2012; 31(6): 1156–1166. DOI: 10.1377/hlthaff.2012.0393 22665827

[5] Hong, CS, Siegel, AL and Ferris, TG. Caring for high-need, high-cost patients: What makes for a successful care management program. Issue Brief (Commonw Fund). 2014; 19(9): 1–19. DOI: 10.15868/socialsector.25007 25115035

[6] Long, P, Abrams, MK, Milstein, A, et al. Effective Care for High-Need Patients: Opportunities for Improving Outcomes, Value, and Health. Washington, DC: National Academy of Medicine; 2017. 37748007

[7] Association AD. 11. Older Adults: Standards of Medical Care in Diabetes—2018. Diabetes care. 2018; 4(Supplement 1): S119–S125. DOI: 10.2337/dc18-S011 29222382

[8] Goetzel, RZ, Staley, P, Ogden, L, et al. A framework for patient-centered health risk assessments: Providing health promotion and disease prevention services to Medicare beneficiaries; 2011.

[9] Centers for Medicare and Medicaid Services. The ABCs of the Annual Wellness Visit Medicare Learning Network, ed2017 Available at: https://www.cms.gov/Outreach-and-Education/Medicare-Learning-Network-MLN/MLNProducts/downloads/AWV_chart_ICN905706.pdf. Accessed 10/25/2018.

[10] Quan, H, Sundararajan, V, Halfon, P, et al. Coding algorithms for defining comorbidities in ICD-9-CM and ICD-10 administrative data. Medical care. 2005; 1130–1139. DOI: 10.1097/01.mlr.0000182534.19832.83 16224307

[11] Linzer, DA and Lewis, JB. poLCA: An R package for polytomous variable latent class analysis. Journal of Statistical Software. 2011; 42(10): 1–29. DOI: 10.18637/jss.v042.i10

[12] Eshghi, A, Haughton, D, Legrand, P, Skaletsky, M and Woolford, S. Identifying groups: A comparison of methodologies. Journal of Data Science. 2011; 9(2): 271–291.

[13] Bates, DW, Saria, S, Ohno-Machado, L, Shah, A and Escobar, G. Big data in health care: Using analytics to identify and manage high-risk and high-cost patients. Health Affairs. 2014; 33(7): 1123–1131. DOI: 10.1377/hlthaff.2014.0041 25006137

[14] Bayliss, E, Tabano, H, Gill, T, et al. Data Management for Applications of Patient Reported Outcomes. 2018; 6(1): 1–8. 10.5334/egems.201 PMC5983068 29881763

[15] Bayliss, EA, Powers, JD, Ellis, JL, Barrow, JC, Strobel, M and Beck, A. Applying Sequential Analytic Methods to Self-Reported Information to Anticipate Care Needs. eGEMs. 2016; 4(1). DOI: 10.13063/2327-9214.1258 PMC4975568 27563684

