# Supplementary material for: Using Self-Reported Data to Segment Older Adult Populations with Complex Care Needs
Source: EGEMS (Wash DC). 2019 Apr 12;7(1):12. doi: 10.5334/egems.275 (PMC6484372; doi:10.5334/egems.275)
Supplement: Appendix A. — Full 14 cluster solution. [file egems-7-1-275-s1.pdf]

Appendix A. Full 14 cluster solution[1]

Input Variable	Total Sample N=9617		Cluster #						
			1 N=936	2 N=485	3 N=278	4 N=1197	5 N=560	6 N=408	7 N=2107
	N (%)	95% CI	N (9.7%)[2]	N (5.0%)[2]	N (2.9%)[2]	N (12.4%)[2]	N (5.8%)[2]	N (4.2%)[2]	N (21.9%)[2]
Fair or Poor General/Physical Health	1599 (16.6)	14.8-18.4	75 (8.0)	157 (32.4)	230 (82.7)	69 (5.8)	40 (7.1)	106 (26.0)	74 (3.5)
Fair or Poor Mental Health	937 (9.7)	7.8-11.6	27 (2.9)	17 (3.5)	149 (53.6)	43 (3.6)	62 (11.1)	32 (7.8)	44 (22.1)
Positive on PHQ-2	887 (9.2)	7.3-11.1	22 (2.4)	63 (13.0)	215 (77.3)	36 (3.0)	20 (3.6)	43 (10.5)	45 (2.1)
Positive on GAD-2	240 (2.5)	0.5-4.5	8 (0.9)	12 (2.5)	59 (21.2)	11 (0.9)	6 (1.1)	6 (1.5)	12 (0.6)
Pain Interferes with Activities	3567 (37.1)	35.5-38.7	151 (16.1)	393 (81.0)	259 (93.2)	180 (15.0)	199 (35.5)	400 (98.0)	274 (13.0)
Fairly or Very Bad Sleep Quality	1617 (16.8)	15.0-18.6	60 (6.4)	81 (6.7)	194 (69.8)	85 (7.1)	78 (13.9)	408 (100.0)	126 (6.0)
Often or Always Lonely/Isolated	276 (2.9)	0.9-4.9	4 (0.4)	14 (2.9)	60 (21.6)	19 (1.6)	14 (2.5)	14 (3.4)	14 (0.7)
Fall in Past 12 Months	2407 (25.0)	23.3-26.7	116 (12.4)	344 (70.9)	158 (56.8)	119 (9.9)	265 (47.3)	67 (16.4)	217 (10.3)
Problem with Balance or Walking	3984 (41.4)	39.9-42.9	155 (16.6)	442 (91.1)	258 (92.8)	93 (7.8)	443 (79.1)	128 (31.4)	63 (3.0)
Problems with Hearing	4468 (46.5)	45.0-48.0	384 (41.0)	191 (39.4)	181 (65.1)	387 (32.3)	439 (78.4)	190 (46.6)	683 (32.4)
Tooth/Mouth Problems	1002 (10.4)	8.5-12.3	57 (6.1)	21 (4.3)	102 (36.7)	87 (7.3)	63 (11.3)	38 (9.3)	64 (3.0)
Accidentally Leaked Urine	4244 (44.1)	42.6-45.6	233 (24.9)	372 (76.7)	209 (75.2)	275 (23.0)	404 (72.1)	194 (47.5)	544 (25.8)
Problems with Memory	1670 (17.4)	15.6-19.2	71 (7.6)	69 (14.2)	145 (52.2)	79 (6.6)	560 (100.0)	40 (9.8)	88 (4.2)
Difficulty Dressing/Using Toilet/Bathing/Getting In & Out of Bed	1008 (10.5)	8.6-12.4	22 (2.4)	296 (61.0)	197 (70.9)	14 (1.2)	23 (4.1)	23 (5.6)	28 (1.3)
Difficulty Eating	371 (3.9)	1.9-5.9	3 (0.3)	44 (9.1)	89 (32.0)	9 (0.8)	10 (1.8)	9 (2.2)	12 (0.6)
Difficulty Taking Medicines/Managing $	496 (5.2)	3.2-7.2	9 (1.0)	22 (4.5)	74 (26.6)	12 (1.0)	22 (3.9)	12 (2.9)	8 (0.4)
Difficulty Shopping/Household Activities	1903 (19.8)	18.0-21.6	0 (0.0)	447 (92.2)	260 (93.5)	6 (0.5)	60 (10.7)	55 (13.5)	0 (0.0)
Tobacco Use	628 (6.5)	4.6-8.4	57 (6.1)	24 (4.9)	27 (9.7)	120 (10.0)	24 (4.3)	25 (6.1)	80 (3.8)
Physically Inactive	3139 (32.6)	31.0-34.2	143 (15.3)	283 (58.4)	225 (80.9)	229 (19.1)	130 (23.2)	70 (17.2)	0 (0.0)
Not Always Enough Money for Food	368 (3.8)	1.8-5.8	15 (1.6)	17 (3.5)	44 (15.8)	37 (3.1)	17 (3.0)	13 (3.2)	40 (1.9)
Do Not Live Independently	2222 (23.1)	21.3-24.9	936 (100.0)	152 (31.3)	62 (22.3)	0 (0.0)	110 (19.6)	98 (24.0)	0 (0.0)
No Advance Directive	2916 (30.3)	28.6-32.0	333 (35.6)	121 (24.9)	104 (37.4)	1197 (100.0)	117 (20.9)	147 (36.0)	0 (0.0)
**Descriptive variable**									
Education									
< HS Graduate	703 (7.3)	5.4-9.2	54 (5.8)	35 (7.2)	40 (14.4)	106 (8.9)	45 (8.0)	36 (8.8)	81 (3.8)
HS/Some College	4868 (50.6)	49.2-52.0	453 (48.4)	251 (51.8)	166 (59.7)	669 (55.9)	265 (47.3)	217 (53.2)	918 (43.6)
College Graduate or More	3349 (34.8)	33.2-36.4	391 (41.8)	148 (30.5)	47 (16.9)	381 (31.8)	222 (39.6)	133 (32.6)	898 (42.6)
Missing	697 (7.2)	5.3-9.1	38 (4.1)	51 (10.5)	25 (9.0)	41 (3.4)	28 (5.0)	22 (5.4)	210 (10.0)
Marital Status									

Married/Committed Relationship	5537 (57.6)	56.3-58.9	699 (74.7)	265 (54.6)	138 (49.6)	746 (62.3)	345 (61.6)	247 (60.5)	1224 (58.1)
Single/Divorced/Separated/Widowed	3478 (36.2)	34.6-37.8	208 (22.2)	178 (36.7)	121 (43.5)	419 (35.0)	189 (33.8)	144 (35.3)	692 (32.8)
Missing	602 (6.3)	4.4-8.2	29 (3.1)	42 (8.7)	19 (6.8)	32 (2.7)	26 (4.6)	17 (4.2)	191 (9.1)
History of cancer	2836 (29.5)	27.8-31.2	269 (28.7)	125 (25.8)	63 (22.7)	325 (27.2)	179 (32.0)	116 (28.4)	706 (33.5)
Age at MTHA									
63-69	2273 (23.6)	21.9-25.3	265 (28.3)	75 (15.5)	59 (21.2)	431 (36.0)	95 (17.0)	131 (32.1)	520 (24.7)
70-79	4478 (46.6)	45.1-48.1	463 (49.5)	188 (38.8)	128 (46.0)	603 (50.4)	257 (45.9)	196 (48.0)	1072 (50.9)
80+	2866 (29.8)	28.1-31.5	208 (22.2)	222 (45.8)	91 (32.7)	163 (13.6)	208 (37.1)	81 (19.9)	515 (24.4)
Female gender	5043 (52.4)	51.0-53.8	361 (38.6)	327 (67.4)	171 (61.5)	567 (47.4)	267 (47.7)	210 (51.5)	1031 (48.9)
Quan Elixhauser score Mean (SD)	4.34 (2.74)		3.98 (2.59)	5.52 (2.92)	5.81 (2.65)	3.63 (2.51)	4.43 (2.71)	4.57 (2.83)	3.63 (2.51)
Median (25%, 75%)	4 (0, 17)		4 (0, 15)	5 (0, 14)	6 (0, 13)	3 (0, 15)	4 (0, 14)	4 (0, 15)	3 (0, 14)
Hospital utilization									
ED Visit(s) 0/1	2029 (21.1)	19.3-22.9	165 (17.6)	141 (29.1)	88 (31.7)	206 (17.2)	135 (24.1)	91 (22.3)	373 (17.7)
Inpatient Admission(s) 0/1	1933 (20.1)	18.3-21.9	177 (18.9)	141 (29.1)	60 (21.6)	193 (16.1)	116 (20.7)	100 (24.5)	351 (16.7)
Observation Admission(s) 0/1	833 (8.7)	6.8-10.6	73 (7.8)	57 (11.8)	35 (12.6)	59 (4.9)	29 (5.2)	38 (9.3)	150 (7.1)

**Input Variable**	**Total Sample**		**Cluster #**						
			**8 N=441**	**9 N=289**	**10 N=515**	**11 N=773**	**12 N=372**	**13 N=345**	**14 N=911**
	**N (%)**	**95% CI**	**N (4.6%)[2]**	**N (3.0%)[2]**	**N (5.4%)[2]**	**N (8.0%)[2]**	**N (3.9%)[2]**	**N (3.6%)[2]**	**N (9.5%)[2]**
Fair or Poor General/Physical Health	1599 (16.6)	14.8-18.4	74 (16.8)	109 (37.7)	162 (31.5)	72 (9.3)	120 (32.3)	242 (70.1)	69 (7.6)
Fair or Poor Mental Health	937 (9.7)	7.8-11.6	17 (3.9)	141 (48.8)	9 (1.7)	27 (3.5)	29 (7.8)	329 (95.4)	11 (1.2)
Positive on PHQ-2	887 (9.2)	7.3-11.1	23 (5.2)	55 (19.0)	41 (8.0)	46 (6.0)	50 (13.4)	202 (58.6)	26 (2.9)
Positive on GAD-2	240 (2.5)	0.5-4.5	10 (2.3)	15 (5.2)	7 (1.4)	6 (0.8)	7 (1.9)	70 (20.3)	11 (1.2)
Pain Interferes with Activities	3567 (37.1)	35.5-38.7	120 (27.2)	123 (42.6)	504 (97.9)	32 (4.1)	252 (67.7)	241 (69.9)	439 (48.2)
Fairly or Very Bad Sleep Quality	1617 (16.8)	15.0-18.6	30 (6.8)	43 (14.9)	120 (23.3)	69 (8.9)	91 (24.5)	166 (48.1)	66 (7.2)
Often or Always Lonely/Isolated	276 (2.9)	0.9-4.9	7 (1.6)	18 (6.2)	8 (1.6)	13 (1.7)	7 (1.9)	75 (21.7)	9 (1.0)
Fall in Past 12 Months	2407 (25.0)	23.3-26.7	52 (11.8)	146 (50.5)	95 (18.4)	182 (23.5)	132 (35.5)	112 (32.5)	402 (44.1)
Problem with Balance or Walking	3984 (41.4)	39.9-42.9	140 (31.7)	244 (84.4)	425 (82.5)	258 (33.4)	304 (81.7)	226 (65.5)	805 (88.4)
Problems with Hearing	4468 (46.5)	45.0-48.0	158 (35.8)	170 (58.8)	237 (46.0)	395 (51.1)	238 (64.0)	220 (63.8)	595 (65.3)
Tooth/Mouth Problems	1002 (10.4)	8.5-12.3	29 (6.6)	69 (23.9)	0 (0.0)	27 (3.5)	372 (100.0)	45 (13.0)	28 (3.1)
Accidentally Leaked Urine	4244 (44.1)	42.6-45.6	141 (32.0)	195 (67.5)	342 (66.4)	342 (44.2)	221 (59.4)	170 (49.3)	602 (66.1)
Problems with Memory	1670 (17.4)	15.6-19.2	42 (9.5)	247 (85.5)	38 (7.4)	42 (5.4)	59 (15.9)	190 (55.1)	0 (0.0)
Difficulty Dressing/Using Toilet/Bathing/Getting In & Out of Bed	1008 (10.5)	8.6-12.4	53 (12.0)	183 (63.3)	24 (4.7)	13 (1.7)	53 (14.2)	25 (7.2)	54 (5.9)

Difficulty Eating	371 (3.9)	1.9-5.9	23 (5.2)	95 (32.9)	9 (1.7)	6 (0.8)	47 (12.6)	8 (2.3)	7 (0.8)
Difficulty Taking Medicines/Managing $	496 (5.2)	3.2-7.2	48 (10.9)	232 (80.3)	8 (1.6)	5 (0.6)	20 (5.4)	18 (5.2)	6 (0.7)
Difficulty Shopping/Household Activities	1903 (19.8)	18.0-21.6	441 (100.0)	273 (94.5)	131 (25.4)	1 (0.1)	155 (41.7)	50 (14.5)	24 (2.6)
Tobacco Use	628 (6.5)	4.6-8.4	37 (8.4)	11 (3.8)	36 (7.0)	56 (7.2)	67 (18.0)	28 (8.1)	36 (4.0)
Physically Inactive	3139 (32.6)	31.0-34.2	209 (47.4)	177 (61.2)	512 (99.4)	773 (100.0)	237 (63.7)	151 (43.8)	0 (0.0)
Not Always Enough Money for Food	368 (3.8)	1.8-5.8	25 (5.7)	16 (5.5)	18 (3.5)	28 (3.6)	48 (12.9)	32 (9.3)	18 (2.0)
Do Not Live Independently	2222 (23.1)	21.3-24.9	157 (35.6)	209 (72.3)	130 (25.2)	62 (8.0)	84 (22.6)	81 (23.5)	141 (15.5)
No Advance Directive	2916 (30.3)	28.6-32.0	139 (31.5)	60 (20.8)	163 (31.7)	100 (12.9)	193 (51.9)	137 (39.7)	105 (11.5)
**Descriptive variable**									
Education									
< HS Graduate	703 (7.3)	5.4-9.2	40 (9.1)	28 (9.7)	46 (8.9)	66 (8.5)	49 (13.2)	45 (13.0)	32 (3.5)
HS/Some College	4868 (50.6)	49.2-52.0	225 (51.0)	123 (42.6)	314 (61.0)	445 (57.6)	211 (56.7)	188 (54.5)	423 (46.4)
College Graduate or More	3349 (34.8)	33.2-36.4	103 (23.4)	82 (28.4)	133 (25.8)	238 (30.8)	77 (20.7)	91 (26.4)	405 (44.5)
Missing	697 (7.2)	5.3-9.1	73 (16.6)	56 (19.4)	22 (4.3)	24 (3.1)	35 (9.4)	21 (6.1)	51 (5.6)
Marital Status									
Married/Committed Relationship	5537 (57.6)	56.3-58.9	179 (40.6)	107 (37.0)	267 (51.8)	415 (53.7)	183 (49.2)	186 (53.9)	536 (58.8)
Single/Divorced/Separated/Widowed	3478 (36.2)	34.6-37.8	199 (45.1)	134 (46.4)	230 (44.7)	340 (44.0)	159 (42.7)	141 (40.9)	324 (35.6)
Missing	602 (6.3)	4.4-8.2	63 (14.3)	48 (16.6)	18 (3.5)	18 (2.3)	30 (8.1)	18 (5.2)	51 (5.6)
History of cancer	2836 (29.5)	27.8-31.2	112 (25.4)	70 (24.2)	146 (28.3)	245 (31.7)	120 (32.3)	85 (24.6)	275 (30.2)
Age at MTHA									
63-69	2273 (23.6)	21.9-25.3	79 (17.9)	42 (14.5)	95 (18.4)	123 (15.9)	88 (23.7)	102 (29.6)	168 (18.4)
70-79	4478 (46.6)	45.1-48.1	164 (37.2)	84 (29.1)	250 (48.5)	356 (46.1)	168 (45.2)	153 (44.3)	396 (43.5)
80+	2866 (29.8)	28.1-31.5	198 (44.9)	163 (56.4)	170 (33.0)	294 (38.0)	116 (31.2)	90 (26.1)	347 (38.1)
Female gender	5043 (52.4)	51.0-53.8	293 (66.4)	174 (60.2)	347 (67.4)	426 (55.1)	213 (57.3)	168 (48.7)	488 (53.6)
Quan Elixhauser score Mean (SD)	4.34 (2.74)		5.11 (2.77)	5.29 (2.86)	5.06 (2.74)	4.53 (2.72)	5.03 (2.82)	5.06 (2.61)	4.29 (2.73)
Median (25%, 75%)	4 (0, 17)		5 (0, 14)	5 (0, 15)	5 (0, 16)	4 (0, 14)	5 (0, 17)	5 (0, 13)	4 (0, 14)
Hospital utilization									
ED Visit(s) 0/1	2029 (21.1)	19.3-22.9	98 (22.2)	96 (33.2)	119 (23.1)	147 (19.0)	85 (22.8)	98 (28.4)	187 (20.5)
Inpatient Admission(s) 0/1	1933 (20.1)	18.3-21.9	117 (26.5)	72 (24.9)	131 (25.4)	143 (18.5)	64 (17.2)	67 (19.4)	201 (22.1)
Observation Admission(s) 0/1	833 (8.7)	6.8-10.6	48 (10.9)	40 (13.8)	42 (8.2)	74 (9.6)	40 (10.8)	41 (11.9)	107 (11.7)

[1] Yellow shading indicates cluster has a proportion of the input variable that is greater than the 95% CI for the population average and the 1st or 2nd highest proportion of all clusters. Blue shading indicates the cluster has a proportion of the input variable that is less than the 95% CI for the population average and the 1st or 2nd lowest proportion of all clusters.

[2] Row percentages this row only. All other percentages in the table reflect proportions of columns.
